# The Predictive Role of Lymphocyte-to-Monocyte Ratio in Acute Kidney Injury in Acute Debakey Type I Aortic Dissection

**DOI:** 10.3389/fsurg.2021.704345

**Published:** 2021-08-11

**Authors:** Xiaochun Ma, Shanghao Chen, Yan Yun, Diming Zhao, Jinzhang Li, Zezhong Wu, Yanwu Liu, Hechen Shen, Huibo Ma, Zhengjun Wang, Chengwei Zou, Haizhou Zhang

**Affiliations:** ^1^Department of Cardiovascular Surgery, Shandong Provincial Hospital, Cheeloo College of Medicine, Shandong University, Jinan, China; ^2^Department of Cardiovascular Surgery, Shandong Provincial Hospital Affiliated to Shandong First Medical University, Jinan, China; ^3^Department of Radiology, Qilu Hospital of Shandong University, Jinan, China; ^4^Qingdao University Medical College, Qingdao University, Qingdao, China

**Keywords:** lymphocyte-to-monocyte ratio, acute kidney injury, acute Debakey type I aortic dissection, predictive model, risk factor

## Abstract

**Background:** The post-operative acute kidney injury (AKI) represents a common complication in the Acute Debakey Type I Aortic Dissection (ADTIAD) and predicts a poorer prognosis. The clinical evidence is scarce supporting the predictive value of the pre-operative lymphocyte-to-monocyte ratio (LMR) in post-operative AKI in ADTIAD.

**Methods:** In this retrospective cohort study, 190 consecutive patients with ADTIAD enrolled for surgical treatment between January 1, 2013, and December 31, 2018. The diagnosis of AKI followed the Kidney Disease: Improving Global Outcomes guidelines (KDIGO). Pre-operative LMR and other possible risk factors were analyzed for their prognostic value in the post-operative AKI in ADTIAD.

**Results:** The subjects were assigned to the low-LMR and high-LMR groups according to the median value of pre-operative LMR. For post-operative AKI, the incidence and the severity in the low-LMR group were statistically different from that of the high-LMR group. Besides, the lower LMR was statistically associated with the more extended ICU stay and intubation time and higher incidences of ischemic stroke and in-hospital mortality. Additionally, in the multivariable analysis, the pre-operative LMR was an independent predictor for post-operative AKI in ADTIAD. A predictive model for post-operative AKI in ADTIAD was established incorporating LMR.

**Conclusions:** LMR is an independent prognostic indicator incorporated into the predictive model with other risk factors to predict the post-operative AKI in ADTIAD.

## Introduction

Acute Debakey Type I Aortic Dissection (ADTIAD) is widely accepted as an urgent and catastrophic aortic pathology in which aortic lesion extends from aortic root, aortic arch to even distal abdominal aorta (1). Several decades have witnessed the fantastic breakthroughs of surgical strategy, cardiopulmonary bypass (CPB), anesthesia, and intensive care in the treatment of ADTIAD (2). Although the short- and long-term outcomes of ADTIAD are currently favorable worldwide, the post-operative complications are still enormous medical challenges for cardiovascular surgeons (3).

Acute kidney injury (AKI) remains a frequent complication following surgical intervention in ADTIAD. The documented incidence of AKI after thoracic aortic surgery ranges from 17 to 68%, depending on the definition of AKI and surgical options (4–6). It is tightly correlated with a dismal prognosis and leads to a higher incidence of mortality and morbidity (7). Previous evidence supported that AKI is an independent predictor of in-hospital mortality after cardiothoracic surgery (8). And even mild AKI increases 30- and 90 day- mortality, morbidity, and cost (9, 10). What is worse, the mechanism of post-operative AKI is still elusive. Additionally, no effective therapy has been developed for AKI through continuous renal replacement therapy (CRRT) serves as a plausible therapeutic option (11).

Increasing evidence has shown that peripheral blood cells and relevant ratios are related to the inflammation as well as AKI after cardiovascular surgery, such as platelet-to-lymphocyte ratio (PLR) and neutrophil-to-lymphocyte ratio (NLR) (12, 13). Lymphocyte-to-monocyte ratio (LMR), calculated from the lymphocyte and monocyte counts of peripheral blood, has gained increasing attention for its independent prognostic value of clinical outcomes in multiple malignant cancers (14–18). A lower LMR seems to be associated with increased systemic inflammation and decreased survival in malignancies (19). Of note, the prevailing opinion advocates that the inflammatory response is aberrantly enhanced in ADTIAD (20, 21). And the intensive shock from the inflammatory response is among one of the well-documented theories underlying the occurrence of AKI (22, 23). In the prevention and treatment of AKI, anti-inflammatory therapy has shown feasible effects in contemporary clinical practice (24). And one aspect of CRRT lies in eliminating the excessive inflammatory mediators in patients with ADTIAD (25). Therefore, the LMR as a useful inflammatory biomarker is very likely to function as a valuable predictive indicator of post-operative AKI in ADTIAD, tightly associated with systemic inflammation. To our best knowledge, such investigation of the relation of LMR and AKI in ADTIAD has been scarcely reported. This retrospective cohort study's primary objective was to confirm whether LMR was an independent predictive indicator of post-operative AKI in ADTIAD.

## Materials and Methods

### Participants and Study Design

This study was a single-center retrospective cohort study. The Ethics Committee approved it of Shandong Provincial Hospital affiliated to Shandong First Medical University. Due to its retrospective design, the written informed consent had to be waived. The study was performed following the Good Clinical Practice (GCP) and principles of the Declaration of Helsinki.

From January 1, 2013, to December 31, 2018, 214 patients were successively enrolled in the study. The period of this study was chosen based on related funding, which spanned roughly a similar period. The subjects were recruited in the study who satisfied the following criteria: (A) older than 18 years; (B) diagnosed as ADTIAD; (C) undergoing the ascending and total arch replacement (TAR) combined with the frozen elephant trunk (FET) technique. The subjects were removed if the exclusion criteria were met:

- Who died pre-operatively or within 72 h after operations;
- Whose pre-operative and intraoperative data were insufficient;
- Who was pre-operatively diagnosed as renal insufficiency with elevated serum creatinine (SCR), oliguria, anuria, or use of CRRT;
- Who had a shock, limb ischemia, and gastrointestinal ischemia and spinal cord ischemia.

All the operations of TAR with FET were performed by one surgical team.

### Definition of Post-operative AKI in ADTIAD and Post-operative Outcomes

The post-operative AKI was diagnosed in line with the modified Kidney Disease: Improving Global Outcomes guidelines (KDIGO) (26) (Table 1). The patients were subsequently categorized according to their highest levels of the post-operative SCR.

**Table 1:** Stage of post-operative AKI in ADIAD using modified KDIGO criteria.

**Stage**	**Serum creatinine increase**
1	1.5~1.9 times baseline or ≥0.3 mg/dL (26.5 umol/L) increase in serum creatinine
2	2.0~2.9 times baseline in serum creatinine
3	≥3.0 times baseline or ≥4.0 mg/dL (353.6 umol/L) increase in serum creatinine or initiation of renal replacement therapy

The primary post-operative outcome of this study was post-operative AKI. And other post-operative outcomes were the incidences of CRRT, redo surgery and in-hospital mortality, and the exact length of ICU stay and intubation time.

### Calculation of Pre-operative LMR and Grouping by Pre-operative LMR

Pre-operative LMR was calculated from the lymphocyte and monocyte counts in the latest pre-operative blood routine test (LMR = lymphocyte count divided by monocyte count). The cut-off LMR value was calculated from a receiver operating characteristic (ROC) curve on the occurrence of post-operative AKI in ADTIAD. When the Youden Index (Youden Index = sensitivity + specificity-1) of the ROC curve reached its maximum value, the corresponding LMR value was determined as the cut-off value. Then the included subjects were accordingly grouped as a low-LMR group (with their LMR values less than cut-off LMR) and high-LMR group (with their LMR values more than cut-off LMR).

### Anesthesia and Surgical Procedures

Under general anesthesia with endotracheal intubation, a standard median sternotomy was performed. Intravenous anesthetics (propofol with remifentanil) and inhalation agents (sevoflurane or isoflurane) with rocuronium were used for anesthesia. For CPB details, arterial cannulation was routinely positioned in the right axillary artery, innominate artery, or femoral artery; venous cannulation was normally bicaval through the right atrium. And CPB was instituted with a flow of 2.2–2.5 L/min/m^2^. For myocardial protection, cold blood cardioplegia was perfused into the left and right coronary arteries. TAR combined with the FET technique was performed under deep or moderate hypothermic circulatory arrest (DHCA or MHCA). In addition, selective antegrade cerebral perfusion (SACP) *via* the right axillary artery or innominate artery was used for cerebral protection during DHCA or MHCA. Besides, in core cooling, concomitant cardiac procedures were performed if necessary, such as aortic valve replacement or repair and ascending aorta replacement. There were no significant changes in anesthesia, surgical techniques, and perioperative management during the study interval.

### Statistical Analysis

All statistical analysis was carried out with the aid of SPSS Statistics 25.0. Continuous variables were expressed as mean ± standard deviation (SD) if data is normally distributed or as median (Quartile Deviation) if information is not consistent with the normal distribution. Categorical variables were present as frequencies (*n*) with percentages (%). For analyzing continuous variables, the Student' *t*-test was used when the normal distribution was conformed. Otherwise, the non-parametric Mann-Whitney *U*-test was applied if a skewed distribution was met. For categorical variables, chi-square or Fisher's exact-test was selected. The binary logistic regression analysis was employed for the univariable and multivariable analyses. When the efforts were made to construct the multivariable predictive model, all candidate variables derived from the univariable analysis (with a *p*-valve < 0.1) and those possible predictive variables were selected. Besides, the receiver operating curve (ROC) and the Hosmer-Lemeshow test were used to further evaluate the predictive model. A two-sided *p*-valve < 0.05 was considered statistically significant.

## Results

### Patient Characteristics

**Figure 1:**
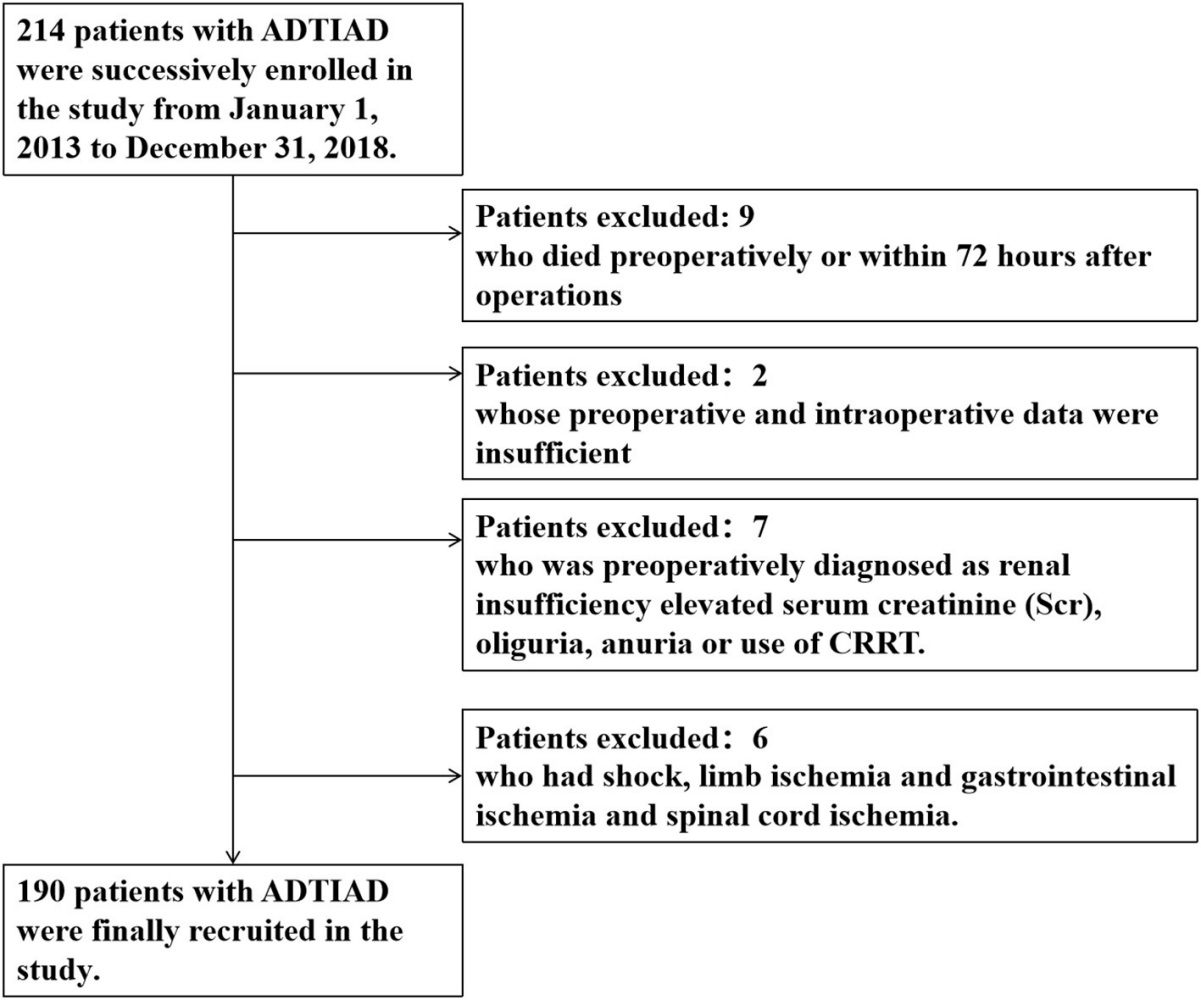
Flow diagram of exclusion and enrollment of study patients. Describes the exclusion and enrollment of study patients.

From January 1, 2013, to December 31, 2018, 214 patients were successively included in the study. Nine patients were excluded from the study who died pre-operatively or within 72 h after operations; 2 patients excluded whose pre-operative and intraoperative data were insufficient; 7 patients excluded who was pre-operatively diagnosed as renal insufficiency with elevated SCR, oliguria, anuria, or use of CRRT; and 6 patients excluded who had a shock, limb ischemia, gastrointestinal ischemia and spinal cord ischemia (Figure 1). Finally, 190 patients with ADTIAD were recruited in the study, of which 65.8% were male patients with a median age of 47.0 (12.3) years. Pre-operatively, the median SCR was 72.3 (28.2) μmol/L, and the median estimated eGFR was 99.3 (23.8) ml/min/1.73 m^2^. The pre-operative median LVEF was 60.0% (2.0%). 31.6% of patients were smokers, and 72.1% had hypertension. Other significant comorbidities consisted of diabetes (3.2%), peripheral vascular disease (7.4%), and chronic pulmonary disease (1.1%). Five patients (2.6%) had a previous history of cardiovascular surgery. Five patients (2.6%) had Marfan syndrome. Eight cases (4.2%) underwent the Bentall procedure, eight subjects (4.2%) underwent the modified Cabrol surgery, seven patients (3.7%) had CABG, and 18 patients (9.5%) underwent valve replacement or shaping. The median aortic cross-clamp time was 98.0 (26.2) min and the median CPB time was 212.0 (43.3) min. The median duration of DHCA or MHCA was 26.0 (12.4) min. After the operation, 131 (68.9%) cases were diagnosed as AKI, 30 patients (15.8%) were intubated for more than 5 days, and 68 patients (35.8%) stayed in the ICU for more than 7 days. The ischemic stroke occurred in 5 (2.6%) patients, and 15 (7.9%) patients underwent secondary surgery. Eleven patients (5.8%) received CRRT, and 11 patients (5.8%) died during hospitalization. Only 21 (11.1%) patients underwent emergent surgery while the others received elective surgery, and the median time gap from admission to surgery was 3.7 (1.3) days.

### Correlation Between LMR and AKI as Well as Other Post-operative Outcomes

#### AKI

**Figure 2:**
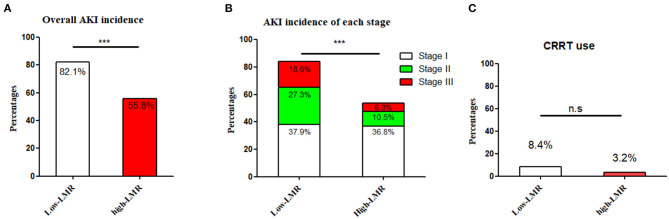
The incidence and severity of post-operative AKI in the low-LMR group and high-LMR group in ADTIAD. Depicts the incidence of AKI and severity (stages 1, 2, and 3) in the low-LMR group and high-LMR group in ADTIAD. **(A)** Incidence of AKI in the low-LMR group and high-LMR group in ADTIAD. **(B)** Severity of AKI (stages 1, 2, and 3) in the low-LMR group and high-LMR group in ADTIAD. **(C)** CRRT use in the low-LMR group and high-LMR group in ADTIAD. AKI, Acute Kidney Injury; LMR, Lymphocyte-to-Monocyte Ratio; CRRT, Continuous Renal Replacement Therapy. ***P < 0.001, n.s., no statistical difference.

The pre-operative LMR was ordered from low to high, in which the median value was 1.51 (1.7). The cut-off value of LMR was calculated from a ROC curve on the occurrence of AKI, which was 1.47 (When the Youden Index reached its maximum value: Youden Index = 0.378, AUC = 0.68, *P*-value < 0.001). Because it was very close to the median value of LMR, thus the cut-off value was eventually decided as 1.51. Accordingly, the subjects were assigned into the low-LMR (*n* = 95) and high-LMR group (*n* = 95). The detailed information of the two groups was summarized in Table 2. For post-operative AKI, the incidence in the low-LMR group was statistically higher than that of the high-LMR group (82.1 vs. 55.8%, *p* < 0.001) (Figure 2A). When AKI was graded, the percentages of stage 1, 2, and 3 in the low-LMR group was 37.9, 27.3, and 18.9% while the percentages in the high-LMR group were 36.8, 10.5, and 6.3%. As was shown above, more patients in the low-LMR group reached the stage 2 and 3 than the ones in the high-LMR (*p* < 0.001) (Figure 2B). The median post-operative highest SCR within 7 days was also evidently elevated in the low-LMR group than the high-LMR group (136.1 vs. 110.3, *p* < 0.001). Additionally, the incidence of CRRT use was not statistically different between the two groups (8.4 vs. 3.2, *p* = 0.13) (Figure 2C).

**Table 2:** Clinical characteristics of patients with ADIAD classified by LMR.

	**Low LMR group**	**High LMR group**	***P*-value**
**Patient population (** ***n*** **)**	95	95	
**Demographic data**			
Age (y)	47.0 (17.0)	47.0 (12.3)	0.73
Sex, male (n)	66 (69.5%)	59 (62.1%)	0.28
Height (cm)	170.0 (10.0)	171.0 (10.0)	0.88
Weight (kg)	75.0 (20.5)	75.0 (15.0)	0.91
BMI (kg/m^2^)	26.2 (5.4)	26.42 (4.79)	0.98
**Medical history**			
Systolic blood pressure at admission (mm Hg)	145.0 (29.0)	137.0 (37.3)	0.20
Diastolic blood pressure at admission (mm Hg)	80.0 (17.0)	79.0 (18.0)	0.51
MAP (mm Hg)	99.3 (17.8)	97.8 (22.4)	0.38
Diabetes (*n*)	1 (1.1%)	5 (5.3%)	0.23
Hypertension (*n*)	70 (73.7%)	67 (70.5%)	0.62
Chronic obstructive pulmonary disease (*n*)	1 (1.1%)	1 (1.1%)	1.00
Previous myocardial infarction (*n*)	0 (0.0%)	5 (5.3%)	0.06
Peripheral vascular disease (*n*)	8 (8.4%)	6 (6.3%)	1.00
Smoking (*n*)	33 (34.7%)	27 (28.4%)	0.35
Pregnancy (*n*)	1 (1.1%)	2 (2.1%)	1.00
Marfan syndrome (*n*)	2 (2.1%)	3 (3.2%)	1.00
**Pre**-**operative laboratory tests**			
Hemoglobin (g/L)	127.0 (22.5)	130 (27.5)	0.25
RBC distribution width CV (%)	12.8 (1.1)	13.1 (1.1)	0.04
RBC distribution width SD (fl)	41.5 (3.8)	42.3 (4.4)	0.11
Platelet (10^9^/L)	170.0 (75)	204.5 (73.8)	0.001
Lymphocyte (10^9^/L)	1.1 (0.6)	1.45 (0.9)	<0.001
Monocyte (10^9^/L)	1.1 (0.6)	0.51 (0.3)	<0.001
Neutrophil(10^9^/L)	9.7 (4.5)	6.21 (4.3)	<0.001
**Urinary protein**			
±	16 (16.8%)	17(17.9%)	1.00
+	36 (37.9%)	13 (13.7%)	<0.001
++	2 (2.1%)	4 (4.2%)	0.68
Cystatin C (mg/L)	0.9 (0.4)	1.0 (0.3)	0.006
Retinol binding protein (mg/L)	25.9 (20.8)	31.2 (19.9)	0.03
INR	1.1 (0.1)	1.08 (0.1)	0.03
**Pre-operative renal function**			
Pre-operative SCR (μmol/L)	69.2 (33.9)	74 (28.1)	0.83
eGFR (mL/min/1.73 m^2^)	103.4 (27.3)	98.5 (26.3)	0.58
Dissections involving the renal artery	34 (35.8%)	27 (28.4%)	0.28
**Pre-operative cardiovascular status**			
Previous cardiovascular surgery (*n*)	0 (0.0%)	5 (5.3%)	0.06
LVEF (%)	60.0 (2.0)	60.0 (2.0)	0.18
Left ventricular hypertrophy (*n*)	55 (57.9%)	50 (52.6%)	0.38
NYHA cardiac function grade III or IV (*n*)	6 (6.3%)	12 (12.6%)	0.21
Marfan syndrome (*n*)	2 (2.1%)	3 (3.2%)	1.00
**Intraoperative blood product use**			
Erythrocytes (u)	5.8 (4.0)	4.0 (4.0)	0.005
Fresh frozen plasma (ml)	450.0 (400.0)	400 (200)	0.17
Platelets (109/L)	113 (75)	120.5 (78.5)	0.60
**Surgical details**			
Involving the aortic arch (*n*)	89 (93.7%)	86 (90.5%)	0.37
Involving the descending aorta (*n*)	84 (88.4%)	74 (77.9%)	0.04
CPB duration (min)	208 (43.5)	192 (51.3)	0.002
DHCA or MHCA (*n*)	95 (100.0%)	95 (100.0%)	1.00
Aortic cross-clamp time (min)	102.5 (28.8)	92.5 (23.3)	0.001
Operation time (h)	8.0 (1.5)	7.5 (1.5)	0.001
Circulatory arrest duration (min)	22.0 (14.0)	22.5 (13.0)	0.46
Cardiopulmonary bypass (*n*)	3 (3.2%)	0 (0.0%)	0.25
**Combined surgery**			
CABG (*n*)	3 (3.2%)	3 (3.2%)	1.00
Valvular surgery (*n*)	5 (5.3%)	11 (11.6%)	0.19
**Post-operative blood product use**			
Post-operative highest SCR within 7 days (μmol/L)	136.1 (134.7)	110.3 (60.3)	<0.001
Hemoglobin (g/L), mean ± standard deviation	106.0 (19.0)	103 (19.8)	0.47
Platelet (10^9^/L)	113.0 (75.0)	120.5 (78.5)	0.76
Lymphocyte (10^9^/L)	0.6 (0.5)	0.6 (0.37)	0.53
Monocyte (10^9^/L)	0.7 (0.5)	0.5 (0.5)	0.002
Neutrophil (10^9^/L)	11.8 (8.4)	10.5 (5.3)	0.02
**Outcomes**			
Continuous Renal replacement therapy (*n*)	8 (8.4%)	3 (3.2%)	0.13
Length of ICU stay (day)	6.0 (5.0)	5.0 (3.0)	0.03
>7 d (*n*)	39 (41.1%)	29 (30.5%)	0.13
Intubation time (h)	43.0 (81.5)	20.5 (29.5)	0.001
>5 d (*n*)	18 (18.9%)	12 (12.6%)	0.24
Stroke (*n*)	6 (6.3%)	0 (0.0%)	0.03
Redo surgery (*n*)	11 (11.6%)	4 (4.2%)	0.06
AKI (*n*)	78 (82.1%)	53 (55.8%)	<0.001
In-hospital mortality (*n)*	10 (10.5%)	2 (2.1%)	0.03

*BMI, body mass index; NYHA, New York Heart Association; INR, International Normalized Ratio; SCR, Serum Creatinine; eGFR, Estimated Glomerular Filtration Rate; LVEF, Left Ventricular Ejection Fraction; CPB, Cardiopulmonary Bypass; DHCA, Deep Hypothermia and Circulatory Arrest; MHCA, Median Hypothermia and Circulatory Arrest; CABG, Coronary Artery Bypass Graft*.

*The categorical variables in the table are presented by the number of cases (with percentage) and the continuous variables are expressed by the median (with interquartile range) or mean (with standard deviation)*.

*P-value: Compare the patients with low LMR and high LMR. P-values were the results of unpaired t-test or Mann-Whitney U-test for continuous variables, and χ^2^-test or Fisher's exact-test for categorical variables*.

Limited by the simple size of the ADTIAD cohort, a quartile analysis was conducted to better stratify the patients based on LMR values. Accordingly, the subjects were subdivided evenly into the very low-LMR (*n* = 48), low-LMR (*n* = 47), high-LMR (*n* = 48), and very high-LMR groups (*n* = 47). For post-operative AKI, the incidence in the four groups was statistically significant (89.4 vs. 78.7% vs. 53.2 vs. 58.7%, *p* < 0.001). When AKI was graded, the percentages of stage 1, 2, and 3 in the very low-LMR group was 38.3, 31.9, and 19.1%, in the low-LMR group was 38.3, 21.3, and 19.1%, while the percentages in the high-LMR group were 38.3, 12.8, and 2.1%, in the very high-LMR group was 37.0, 10.9, and 10.9%, and the percentages of stage 2 and 3 were statistically significant among the four groups (*p* < 0.001).

#### Other Post-operative Outcomes

First, the median duration of ICU stay of the low-LMR group was significantly longer than that of the high-LMR group (6.0 vs. 5.0 days, *p* = 0.03). Second, the patients in the low-LMR group had a longer intubation time than the ones in the high-LMR group (43.0 vs. 20.5 h, *p* = 0.001) significantly. Thirds, more patients in the low-LMR group experienced an ischemic stroke event after surgery than the ones in the high-LMR group (6.3 vs. 0%, *p* = 0.03). Fourth, the two groups were similar in the incidences of secondary surgery, and the results were in the border of statistical significance (11.8 vs. 4.3%, *p* = 0.06). Last but not least, the in-hospital mortality was significantly elevated in the low-LMR group than the high-LMR group (10.5 vs. 2.1%, *p* = 0.03).

### Risk Factors of Post-operative AKI in ADTIAD

#### Univariable Analysis of Risk Factors for AKI in ADTIAD

When the univariable analysis of risk factors for AKI in ADTIAD was performed, male gender, weight, pre-operative lymphocyte, monocyte, neutrophil, NLR, LMR, CPB time, operation time, aortic cross-clamp time, and operation time were significantly different between the patients with or without AKI in ADTIAD (Table 3).

**Table 3:** Univariate analysis of risk factors for AKI in ADIAD.

**Variables**	**OR**	**95% CI**	***p*-value**
Age (y)	0.99	0.95–1.03	0.19
Sex, male	2.03	1.03–3.96	0.03
Height	1.09	0.92–1.42	0.27
Weight	1.04	1.01–1.07	0.009
BMI	1.08	0.96–1.21	0.36
Diabetes	1.23	0.37–5.36	0.86
Hypertension	1.37	0.67–3.26	0.36
Smoking	0.89	0.45–1.75	0.74
Chronic obstructive pulmonary disease	2.36	0.15–38.47	0.55
Peripheral vascular disease	0.93	0.28–3.10	0.91
Previous cardiovascular surgery	0.58	0.06–5.28	0.63
Pre-operative hyperlipidemia	1.21	0.57–2.58	0.61
Pre-operative INR	1.33	0.05–33.13	0.86
Pre-operative hemoglobin	1.02	0.97–1.07	0.67
Pre-operative platelet	1.00	0.99–1.00	0.06
Pre-operative lymphocyte	0.61	0.37–1.01	0.05
Pre-operative monocyte	2.64	1.26–5.78	0.01
Pre-operative neutrophil	1.18	1.04–1.38	0.03
NLR	1.16	1.06–1.28	0.002
PLR	1.00	1.00–1.01	0.43
LMR	0.77	0.64–0.93	0.007
Pre-operative SCR > 106 μmol/L	0.63	0.24–1.65	0.35
Pre-operative eGFR	1.04	0.99–1.09	0.93
Pre-operative LVEF grading	1.73	0.90–3.31	0.1
Pre-operative NYHA cardiac function grade III or IV	0.39	0.14–1.03	0.06
Marfan syndrome	0.34	0.12–1.93	0.16
Intraoperative erythrocytes use	1.06	0.92–1.22	0.42
Intraoperative fresh frozen plasma use	1.00	1.00–1.00	0.41
Intraoperative platelets use	1.23	0.79–2.13	0.54
Involving the aortic arch	2.50	0.77–8.12	0.13
Involving the descending aorta	2.25	0.86–4.46	0.07
CPB duration	1.06	1.02–1.10	<0.001
DHCA or MHCA	2.42	0.72–7.20	0.21
Aortic cross-clamp time	1.03	1.00–1.06	0.01
Operation time	1.63	1.26–2.10	<0.001
Combined CABG	2.22	0.26–20.26	0.48
Combined valvular surgery	0.45	0.12–1.26	0.11

*NLR, Neutrophil to Lymphocyte Ratio; PLR, Platelet to Lymphocyte Ratio; LMR, Lymphocyte to Monocyte Ratio; BMI: body mass index; SCR, Serum Creatinine; eGFR, Estimated Glomerular Filtration Rate; LVEF, Left Ventricular Ejection Fraction; CPB, Cardiopulmonary Bypass; DHCA, Deep Hypothermia and Circulatory Arrest; MHCA, Median Hypothermia and Circulatory Arrest; CABG, Coronary Artery Bypass Graft; OR, Odds Ratio; CI; Confidence Interval*.

#### Multivariable Analysis of Risk Factors for AKI in ADTIAD

Multivariable analysis of risk factors for AKI in ADTIAD was carried out by including all candidate variables derived from the univariable analysis and those possible predictive variables (including CPB time, LMR, age, weight, pre-operative platelet, pre-operative neutrophil, pre-operative LVEF grading, pre-operative NYHA cardiac function grade III or IV, Marfan syndrome, involving the aortic arch, involving the descending aorta, aortic cross-clamp time, operation time). The results showed that CPB time [OR 1.06, 95% CI: (1.02–1.10), *p* < 0.001] and pre-operative low-LMR [OR 0.83, 95% CI: (0.71–0.98), *p* = 0.02] independently predicted the occurrence of post-operative AKI (Table 4).

**Table 4:** Multivariate analysis of risk factors for AKI in ADIAD (first).

**Variables**	***B***	**SE**	***p*-value**	**OR**	**95% CI**
CPB duration (min)	0.019	0.005	<0.001	1.06	1.02–1.10
LMR	−0.183	0.081	0.023	0.83	0.71–0.98
Constant	−5.374	1.567	0.001	0.005	

*LMR, Lymphocyte to Monocyte Ratio; CPB, Cardiopulmonary Bypass; OR, Odds Ratio; CI; Confidence Interval; SE, Standard Error*.

Additionally, another multivariable analysis in which the LMR was introduced as a dichotomous variable was carried out by including all candidate variables derived from the univariable analysis and those possible predictive variables (including CPB time, low-LMR, age, weight, pre-operative platelet, pre-operative neutrophil, pre-operative LVEF grading, pre-operative NYHA cardiac function grade III or IV, Marfan syndrome, involving the aortic arch, involving the descending aorta, aortic cross-clamp time, operation time). The results showed that CPB time [OR 1.02, 95% CI: (1.01–1.03), *p* = 0.001] and pre-operative low-LMR [OR 3.66, 95% CI: (1.78–7.57), *p* < 0.001] independently predicted the occurrence of post-operative AKI (Table 5). The result was consistent that the LMR as a binary variable could also independently predict the occurrence of post-operative AKI in ADTIAD.

**Table 5:** Multivariate analysis of risk factors for AKI in ADIAD (second).

**Variables**	***B***	**SE**	***p*-value**	**OR**	**95% CI**
CPB duration (min)	0.017	0.005	0.001	1.02	1.01–1.03
LMR, low-LMR	1.299	0.370	<0.001	3.66	1.78–7.57
Constant	−2.992	0.987	0.002	0.05	

*LMR, Lymphocyte to Monocyte Ratio; CPB, Cardiopulmonary Bypass; OR, Odds Ratio; CI; Confidence Interval; SE, Standard Error*.

#### Predictive Model for Post-operative AKI in ADTIAD

**Figure 3:**
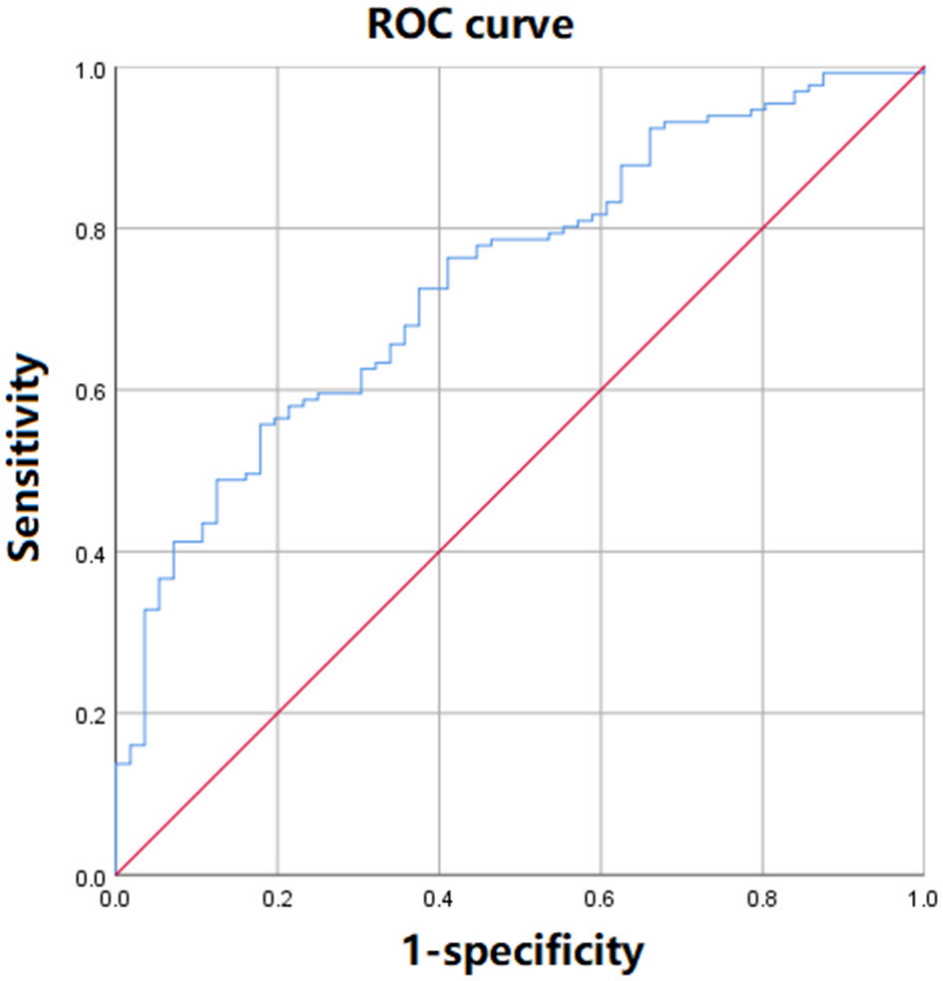
The ROC curve of the predictive model of post-operative AKI incorporating LMR in ADTIAD. This figure shows the ROC curve of predictive models of post-operative AKI incorporating LMR in ADTIAD. AKI, Acute Kidney Injury; LMR, Lymphocyte-to-Monocyte Ratio; ROC, Receiver Operating Characteristic.

For establishing the predictive model for post-operative AKI in ADTIAD, the equation obtained by binary logistic regression analysis was: *Y* = 0.02 × CPB time-0.18 × LMR-5.37. The AUC of ROC curve was 0.74 (*p* < 0.001, 95% CI: 0.67–0.81) (Figure 3). When the maximum value of the Youden index reached 0.38, at this time *Y* = 1.14, the sensitivity of the model was 71.7%, and the specificity was 82.1%.

## Discussion

The occurrence of post-operative AKI markedly deteriorates the short- and long-term prognosis of patients (8, 9, 27), leading to a higher likelihood of mortality and morbidity. Thus, it is a clinical priority to identify the predictive biomarkers or modifiable risk factors in the prevention and treatment of AKI. To the best of our knowledge, the present study is the first one that demonstrated the relationship between the pre-operative LMR and post-operative AKI in ADTIAD. This study presented that the pre-operative LMR possesses an independent predictive value for the post-operative AKI in ADTIAD and that a lower pre-operative LMR is closely associated with the occurrence and severity of post-operative AKI as well as other post-operative outcomes in ADTIAD. Therefore, LMR is considered a novel predictor for post-operative AKI in ADTIAD. Due to the high incidence of post-operative AKI in ADTIAD, the patients with lower pre-operative LMR could be potentially categorized as high-risk populations for clinical trials of renal protective strategies. With the aid of risk stratification of post-operative AKI by pre-operative LMR, early identification of patients at high risk might be achieved, which would lead to the close monitoring and early initiation of efficient preventive and therapeutic strategies. And a more accurate cut-off pre-operative LMR might also be used in the early diagnosis of post-operative AKI in ADTIAD if more prospective studies with larger sample sizes are available to confirm it. And such a predictive cut-off value would be possibly incorporated into a new grading system for post-operative AKI in ADTIAD.

Renal tubular epithelial cells are the primary damaged cells of AKI. Under the damage from ischemia, toxin, inflammation and etc., renal tubular epithelial cells undergo degeneration, apoptosis, necrosis, and shedding (28). And both innate and adaptive immune responses have been involved in the immuno-inflammatory response in the AKI, and their complex regulatory networks are far from clarified (29). The innate immune response system includes neutrophils, monocyte, macrophages, dendritic cells, natural killer cells, and natural killer T cells, while the adaptive immune response system includes T lymphocytes, B lymphocytes, and dendritic cells (30–32). Except for their beneficial role in eliminating endogenous and exogenous antigens, these inflammatory effector cells are excessively activated, releasing a large amount of immuno-inflammatory mediators and resulting in the amplification of immune responses. The consequence is that the renal tubular epithelial cells are further impaired, and their proliferation is markedly prohibited (32). Recently a large number of studies have been conducted on the regulation of inflammatory response in AKI. Overall, direct or indirect suppression of inflammatory response could significantly reduce the degree of renal damage in the AKI animal model, manifested as a relative decline in serum creatinine levels and a reduction in tubular necrosis (31, 33).

LMR is calculated from the exact amounts of lymphocyte and monocyte of peripheral blood into a single index and has been shown as an independent predictive indicator of clinical outcomes in various cancers (14–18). As an inflammation-related indicator, lower LMR appears to be associated with decreased survival and increased recurrence in malignancies (19). Our study manifested that, owing to a lower lymphocyte count and a higher monocyte count, a decreased pre-operative LMR in patients with ADTIAD was inclined to have an increased chance of post-operative AKI. Besides, the lower LMR was statistically associated with the longer ICU stay and intubation time and higher incidences of ischemic stroke and in-hospital mortality. More importantly, the pre-operative LMR was identified as an independent predictor for post-operative AKI in ADTIAD. This was the first report about the correlation between LMR and post-operative AKI and other post-operative outcomes in cardiac surgery. Besides, our model also showed that CPB time is another risk factor for post-operative AKI in ADTIAD, which has also been confirmed in a recent finding (34). Additionally, the results of the diagnostic test suggested that our predictive model for post-operative AKI in ADTIAD has a relatively good predictive ability, with its sensitivity and specificity to be further improved in the future (34). These results are awaiting further verification by large-scale cohort studies in multiple ethnicities in the future.

### Limitations

Several limitations were present, and special attention should be paid to interpret the results in this study. First, the retrospective design inevitably introduced a source of potential bias, and it was a single-center study. Second, the sample size of the ADTIAD cohort was relatively small. Third, LMR might be affected by the confounding variables. Thus, the multiple logistic regression analysis was performed by including them as independent variables. Fourth, the mid-to-long-term outcomes were missing in this study. Fifth, the design of this study did not allow the evaluation of the actual causal relationships between pre-operative LMR and post-operative AKI. And the reported data were mainly descriptive and did not delve into the causes of the association between pre-operative LMR and post-operative AKI.

## Conclusions

A lower LMR has associated higher risk of post-operative AKI, and it has an independent predictive value of post-operative AKI in ADTIAD. In the future, multi-center prospective studies with larger cohorts are warranted to evaluate the results.

## Data Availability Statement

The raw data supporting the conclusions of this article will be made available by the authors, without undue reservation.

## Ethics Statement

The studies involving human participants were reviewed and approved by The Ethics Committee of Shandong Provincial Hospital affiliated to Shandong First Medical University. Written informed consent was not required for this study, in accordance with the local legislation and institutional requirements.

## Author Contributions

XM, HZ, ZWa, and CZ contributed to the conception and design of the study. YY, DZ, HM, and JL organized the database. SC, ZWu, YL, and HS performed the statistical analysis. XM wrote the first draft of the manuscript. XM, SC, YY, and HZ wrote sections of the manuscript. All authors contributed to manuscript revision, read, and approved the submitted version.

## Conflict of Interest

The authors declare that the research was conducted in the absence of any commercial or financial relationships that could be construed as a potential conflict of interest.

## Publisher's Note

All claims expressed in this article are solely those of the authors and do not necessarily represent those of their affiliated organizations, or those of the publisher, the editors and the reviewers. Any product that may be evaluated in this article, or claim that may be made by its manufacturer, is not guaranteed or endorsed by the publisher.
